# Antivenom access impacts severity of Brazilian snakebite envenoming: A geographic information system analysis

**DOI:** 10.1371/journal.pntd.0011305

**Published:** 2023-06-21

**Authors:** Julia Elizabeth Isaacson, Jinny Jing Ye, Lincoln Luís Silva, Thiago Augusto Hernandes Rocha, Luciano de Andrade, Joao Felipe Hermann Costa Scheidt, Fan Hui Wen, Jacqueline Sachett, Wuelton Marcelo Monteiro, Catherine Ann Staton, Joao Ricardo Nickenig Vissoci, Charles John Gerardo

**Affiliations:** 1 Duke University School of Medicine, Durham, North Carolina, United States of America; 2 Department of Emergency Medicine, Duke University School of Medicine, Durham, North Carolina, United States of America; 3 Post-Graduation Program in Biosciences and Physiopathology, State University of Maringá, Maringá, Paraná, Brazil; 4 Duke Global Health Institute, Durham, North Carolina, United States of America; 5 Department of Medicine, State University of Maringá, Maringá, Paraná, Brazil; 6 Antivenom Production Section, Butantan Institute, São Paulo, São Paulo, Brazil; 7 School of Health Sciences, University of Amazonas State, Manaus, Amazonas, Brazil; 8 Tropical Medicine Foundation Dr. Heitor Vieira Dourado, Manaus, Amazonas, Brazil; Centro para el Desarrollo de la Investigación Científica, PARAGUAY

## Abstract

**Background:**

Snakebite envenoming (SBE) is a neglected tropical disease capable of causing both significant disability and death. The burden of SBE is especially high in low- and middle-income countries. The aim of this study was to perform a geospatial analysis evaluating the association of sociodemographics and access to care indicators on moderate and severe cases of SBE in Brazil.

**Methods:**

We conducted an ecological, cross-sectional study of SBE in Brazil from 2014 to 2019 using the open access National System Identification of Notifiable Diseases (SINAN) database. We then collected a set of indicators from the Brazil Census of 2010 and performed a Principal Component Analysis to create variables related to health, economics, occupation, education, infrastructure, and access to care. Next, a descriptive and exploratory spatial analysis was conducted to evaluate the geospatial association of moderate and severe events. These variables related to events were evaluated using Geographically Weighted Poisson Regression. T-values were plotted in choropleth maps and considered statistically significant when values were <-1.96 or >+1.96.

**Results:**

We found that the North region had the highest number of SBE cases by population (47.83/100,000), death rates (0.18/100,000), moderate and severe rates (22.96/100,000), and proportion of cases that took more than three hours to reach healthcare assistance (44.11%). The Northeast and Midwest had the next poorest indicators. Life expectancy, young population structure, inequality, electricity, occupation, and more than three hours to reach healthcare were positively associated with greater cases of moderate and severe events, while income, illiteracy, sanitation, and access to care were negatively associated. The remaining indicators showed a positive association in some areas of the country and a negative association in other areas.

**Conclusion:**

Regional disparities in SBE incidence and rates of poor outcomes exist in Brazil, with the North region disproportionately affected. Multiple indicators were associated with rates of moderate and severe events, such as sociodemographic and health care indicators. Any approach to improving snakebite care must work to ensure the timeliness of antivenom administration.

## Introduction

Snakebite envenoming (SBE) is a neglected tropical disease and a public health problem, especially in the developing world. Globally, there are an estimated 1.8 to 2.7 million SBEs every year and mortality may be as high as 137,880 deaths annually [1]. The burden of SBE is not distributed equally. Rather, this is a disease affecting resource-limited settings in low- and middle-income countries (LMICs) such as Brazil [2].

Snake venom continues to exert its effects until rendered inactive; thus, a delay in the treatment with antivenom increases the chances of complications and even death [3]. Complications of SBE can be severe and include acute renal failure, soft tissue infection and sepsis, central nervous system hemorrhage, compartment syndrome [4]. Neurotoxicity is an important symptom [5]. Antivenom is the mainstay of treatment, and acts by essentially binding to and neutralizing toxins in snake venom [6]. Therefore, improving access to snakebite antivenom is of great importance as most deaths and complications can be prevented by timely antivenom administration [7].

However, barriers to increasing the availability of snakebite care exist in Brazil, especially in the Amazon region where rural populations face serious difficulties to receive adequate treatment. Access to healthcare units often involves long journeys, combined with a low availability of means of transportation and a challenging ecological environment [8]. In the Amazon region, where the distances and transportation difficulties are enormous, accessing healthcare requires significant effort and the process for patients to receive care after SBE can be long and fragmented, marked by changes of means of transport [9]. Formal road systems are limited, highways are poorly maintained, and many towns and villages developed along waterways; thus, a great number of people rely on river transportation [10]. These challenges result in a delay in care, which has been shown to be an independent risk factor for severity and mortality post-SBE [3,11].

Despite a nationalized healthcare system in Brazil, half of the country’s municipalities do not have easy access to emergency care services [12]. In addition, since the healthcare coverage depends on population and the municipalities vary widely in size, there is substantial heterogeneity of care with those who reside in rural communities and in the Amazon region disproportionately affected [12]. Additionally, the heterogeneity of worse SBE outcomes in Brazil could be linked to snake species, their distribution, and distribution of human populations sharing the same habitat [13]. Consequently, this issue can be approached by considering vulnerability to snakebite envenoming as a nexus of ecological contexts and public health weaknesses [14]. Therefore, the aim of this study is to use spatial analysis to evaluate the association between the number of moderate and severe cases of SBE and sociodemographic indicators in Brazil.

## Methods

### Study design

This is an ecological, cross-sectional data study of nationwide SBE from 2014 to 2019 in Brazil from the National System Identification of Notifiable Diseases (SINAN) database. SINAN is an open access database stored at the Information Technology Department of the Brazilian Public Health Care System (DATASUS). We conducted a secondary descriptive and exploratory spatial analysis at the municipality level to evaluate the geospatial association of moderate and severe cases of SBE in Brazil with sociodemographic and healthcare access predictors.

### Study population and location

**Fig 1:**
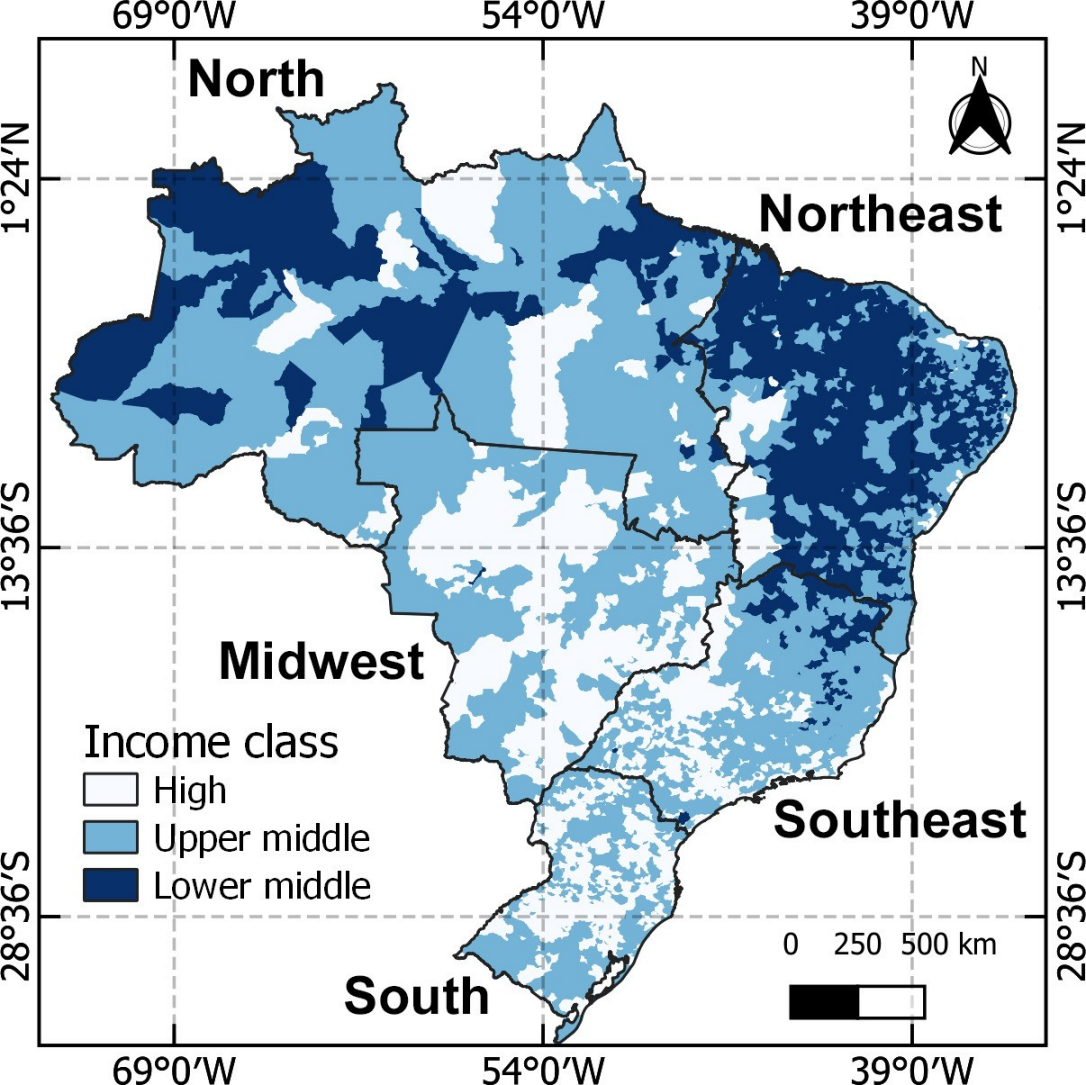
Brazil regions and classification of gross domestic product by municipality. Source for base layer: Instituto Brasileiro de Geografia e Estatística [17].

Brazil is in South America, occupying an area of 8,516,000 square kilometers with a total population of more than 205 million inhabitants distributed through five regions: North, Northeast, Midwest, Southeast and South [15]. Brazil’s gross domestic product has expanded unevenly across different regions, leading to income inequality across the country [16] (Fig 1).

### Outcome definition

We defined SBE as all notified cases of venomous snakebite registered in SINAN (Notifiable Diseases Information System) whether antivenom was administered (either due to lack of availability or dry bites). Additionally, the sum of moderate and severe events is the main variable of interest in this study.

### Data sources, variables, and extraction

Three groups of data were collected: all cases of SBE by municipality in Brazil from 2014 to 2019, the location of health facilities where there is antivenom, and information about socio-demographic aspects at the municipality level. The number of SBE, moderate and severe events cases, the time each patient took to arrive at the health facility, and the number of SBE cases that required antivenom therapy were collected from SINAN and DATASUS (Information Technology Department of the Brazilian Public Health Care System). Information regarding health facilities with antivenom was obtained from the National Poisoning Information System (SINITOX). Hospitals with intensive care unit beds were obtained from the National Register of Health Facilities (CNES). Municipalities in which antivenoms were available were obtained from the Brazilian Ministry of Health database. Sociodemographic indicators were extracted from the 2010 Brazilian Census conducted by the Brazilian Institute for Geography and Statistic (IBGE). The map used for spatial representation was obtained from the IBGE. All this information is openly available at their respective places of access. Data sources are listed below (Table 1).

**Table 1:** Data source and selected variables for the analysis.

Source	Variables	Reference
**DATASUS** **SINAN**	• Frequency of SBE, moderate and severe events by municipalities; • Frequency of SBE related deaths by municipalities; • Frequency of SBE cases receiving antivenom by municipalities; • Frequency of SBE cases reaching the health facility with antivenom within 3 hours or more.	[18]
**CNES—Brazilian Ministry of Health database**	• Hospitals with ICU beds.	[19]
**FIOCRUZ/** **SINITOX**	• Health facilities with antivenom.	[20]
**IBGE**	• Sociodemographic indicators at municipality level.	[21]
	• Geospatial representation of Brazilian municipalities and State.	[17]

DATASUS: Information Technology Department of the Brazilian Public Health Care System; SINAN: National System of Notifiable Disorders; CNES: National Health Facilities Census; IBGE: Brazilian Institute for Geography and Statistic; PNAD: National Household Sample Survey.

### Data analysis

To assess the association between sociodemographic indicators and the incidence of moderate and severe cases caused by SBE, we used an explicative approach based on spatial distribution of SBE cases in order to verify the regional disparities. First, we performed Moran’s I to verify the dependence of spatiality in the distribution of severe SBE outcomes. Values ranging to +1/-1 with p <0.05 indicates the spatial distribution is present, meanwhile 0 is randomness [22]. Next, we built a model with sociodemographic indicators as independent variables and moderate and severe SBE events as dependent using Generalized Linear Models with Poisson regression (GLM). Even though GLM does not take into consideration the spatial influence, it is needed to establish a model without the spatial factor and compare it with other models such as Geographically Weighted Poisson Regression (GWPR) that account for spatial dependency [23]. These two models were compared and the model with the best performance was chosen based on lowest values of Akaike Information Criterion (AIC) and Moran’s residuals, and highest Pseudo R^2^. All analyses in this study were conducted at the municipality level. SBE absolute and relative frequency and its complications, socioeconomic indicators, and healthcare access were summarized using descriptive statistics.

### Sociodemographic indicators

All the sociodemographic indicators were collected from the Atlas of Human Development of Municipalities in Brazil. This open access atlas contains more than 200 indicators from all Brazilian municipalities in the 2010 census. In this study, these indicators were categorized into five major groups: health, economics, occupation, education, and infrastructure (Table 2). For all indicators linked to each aforementioned group, we performed a dimensionality reduction analysis. A principal component analysis (PCA) yielded best results in two dimensions using oblimin rotation in each group. After fitting the models, we created new variables containing the information of indicators of each group, listed in Table 2 [24].

**Table 2:** List of sociodemographic variables used in PCA analysis to create new indicators for spatial analysis.

Group	Indicators selected as input for the PCA analysis	New variables
**Health**	• Life expectancy at birth; • Probability of survival up to 40 and 60 years.	• Life expectancy
	• Total fertility rate; • Child mortality; • Dependency ratio; • Probability of survival up to 40 and 60 years; • Aging rate.	• Young population structure
Economics*	• % of extremely poor (proportion of individuals with per capita household income equal to or less than R$ 70.00 per month from August 2010); • % of extremely poor children (proportion of individuals up to 14 years of age who have a per capita household income equal to or less than R$ 70.00 per month, in reais as of August 2010); • % of poor (proportion of individuals with per capita household income equal to or less than R$ 140.00 per month, in reais as of August 2010); • % of people vulnerable to poverty; • % of income from labor income; • Per capita income; • Average income of employed persons—18 years and over.	• Income
	• Gini Index; • Theil Index—L; • % of income appropriated by the richest 10%; • Ratios between rich and poor population (measure of the degree of inequality existing in the distribution of individuals according to per capita household income. It compares the average per capita income of individuals belonging to the richest tenth of this distribution with the average capita income of individuals belonging to the poorest two-fifths).	• Inequality
**Occupation**	• % of employed workers—18 years old or over; • % of persons employed in the agricultural sector—18 years of age or older; • % of persons employed in the trade sector—18 years of age or older; • % of persons employed in the construction sector—18 years of age or older; • % of persons employed in the mineral extraction sector—18 years of age or older; • % of persons employed in the services sector—18 years of age or older; • % of persons employed in the manufacturing industry—18 years of age or older; • % of employed persons without income—18 years old or over; • % of employed persons with a higher education; • % of public sector workers—18 years of age or older; • % Economically active population;	• Occupation
	• Unemployment rate; • Theil-L index of earnings from work—18 years and over; • % of employees without a formal contract—18 years old or more;	• Formal job
**Education**	• Expectation of years of study; • Illiteracy rate; • Schooling delay; • % of people at school; • % of people graduated from basic, fundamental, and high school, and higher education.	• Schooling
	• % of people that do not attend to school; • Basic attendance rate; • Fundamental frequency rate; • High school frequency rate; • Pre-school attendance rate; • Higher education frequency rate.	• School attendance
**Infrastructure**	• % of the population in households with water; • % of the population in households with bathroom and water; • % of the population in households with garbage collection; • % of people in households with inadequate water supply and sewage; • % of people in households with inadequate walls;	• Sanitation
	• % of people in households without electricity; • % of the population in households with electricity.	• Electricity

*In August 2010, U$ 1 = R$ 1.76

*In December 2022, U$ 1 = R$ 5.21.

### SBE case and healthcare network characterization

To characterize the SBE healthcare network, two markers were selected: [1] the proportion of moderate and severe cases that reached the healthcare facility in more than three hours, and [2] the proportion of cases that required antivenom therapy. For the healthcare network characterization, we calculated the availability of a health facility with ICU beds and antivenom by mapping the hospitals that received antivenom. Data regarding which hospitals are supplied with antivenom was provided by SINITOX. Unfortunately, as there is only a current list of hospitals receiving antivenom, it was not possible to evaluate the availability of antivenom over time. Thus, availability of care was defined by the availability of ICU beds because there is no information regarding the quantity of antivenom available in each health facility, whereas number ICU beds is available in the CNES database and provides a reference to a given health unit’s ability to provide care for a given population. Antivenom distribution is centralized by the Brazilian Ministry of Health and then distributed to 27 federal units. Each federal unit defines which municipalities should have a referral healthcare for antivenom treatment, which is free of charge for the patients. To evaluate the accessibility index to hospitals with antivenom and ICU beds, we used the two-step floating catchment area (2SFCA) method [25,26]. The 2SFCA generates an index of facilities with antivenom weighted by population in two steps. First, a buffer around each facility is created to determine the population covered. A Euclidean 120 km radius threshold was used, approximately equal to two hours of travel time by car or public transportation [27]. The second step consists of summing the initial ratios in overlapped service areas to measure accessibility for a demand location, where residents have access to multiple supply locations. These steps produced an accessibility index of antivenom availability for each municipality in which the coverage is based on Euclidian distances, not taking into consideration the routes and transportation information [27]. A higher accessibility index indicates more hospitals with ICU beds and antivenom available for a given municipality. We used Euclidian distances because the travel times in Brazil are heterogeneous and depend on the type of travel method (walking, car, boat). To date, there is no navigable database to be able to estimate travel time using rivers for the rural areas of Brazil. The Euclidean distance was the best approximation we could use to compare travel time and coverage areas across the Brazilian territory.

### Analytical approach—regression analysis

To assess the association between SBE characteristics and sociodemographic factors, spatial analysis was applied to determine the existence of significant Global Spatial Autocorrelation (Moran’s I) to determine whether the pattern of moderate and severe events cases was distributed randomly or clustered [28,29]. Next, Local Indicator of Spatial Autocorrelation and Getis Ord G analysis were performed to verify formation of clusters and hotspots for the incidence of moderate and severe cases by snakebites envenoming [29,30].

Once the pattern was established, two regression models were tested to determine the best method to explain the relationship of indicators with moderate and severe events. The first model used was the GLM, a linear regression which does not take into account the spatial component, and which builds a single equation to explain the whole phenomenon occurring in the country. Next, as the data showed high spatial autocorrelation of the residuals, GWPR was applied. The GWPR is capable of creating an equation for each municipality and using it with Poisson distribution in order to smooth the inflated number of zero moderate and severe events [31,32]. The regression model that achieved the best performance as defined by the highest adjusted R^2^ value, and the lowest value of AIC and Moran’s I residuals was used to make the study analogies [29]. In this study, the bandwidth used in GWPR was conFigd as adaptive, Poisson distribution on geographical weighting function, and the default method for drop-1 cross-validation.

The predictors were life expectancy, young population structure, income, inequality, schooling, school attendance, sanitation, electricity, occupation, formal work, proportion of cases with more than three hours to reach health care assistance, and healthcare availability. All models were adjusted for population size as a confounder. The outcome indicator was moderate and severe events. All geostatistical analysis was performed on R version 4.1.0 "Camp Pontanezen" using package spdep (1.1–13), spgwr (0.6–34), and GWmodel (2.2–8).

### Thematic map

The results were presented in maps generated using QGIS software version 3.18, with shapefiles obtained from IBGE [17].

### Ethical aspects

This study is based on secondary data freely available and does not contain any sensitive information regarding the population study. Therefore, ethical approval and consent form was not required in accordance with the Brazilian National Guidelines for Research With Human Beings (Resolution 510/2016 of the National Health Council).

## Results

Between 2014 and 2019, there were a total of 137,335 reported cases of SBE in Brazil. Of these, 65,077 resulted in moderate or severe events and 594 deaths. The highest number of cases, moderate and severe events, and deaths by 100,000 population were seen in the North followed by the Midwest. The Southeast had the lowest SBE rates, while the Northeast had the lowest moderate and severe events (Table 3). According to the registry, the majority of patients who experienced SBE received antivenom at some point during their clinical course. However, the North and Northeast regions had a higher proportion of cases requiring more than three hours to reach healthcare followed by the Midwest (Table 3). Notably, the availability of ICU beds in hospitals that also have antivenom is considerably higher in the North and Northeast region in comparison to the rest of the country, in particular the North.

**Table 3:** Descriptive characteristics of snakebite envenomation by region in Brazil from 2014–2019.

Regions	SBE events	Moderate and severe events	Mortality	(%) of cases that took more than 3 hours to reach healthcare assistance	(%) of cases that used antivenom therapy	Median (IQR) of availability of ICU beds and antivenom
**North**	47.83	22.96	0.18	44.11	93.13	1.94 (7.51)
**Northeast**	8.97	3.95	0.05	32.02	74.37	1.95 (1.53)
**Midwest**	15.72	7.63	0.07	19.72	74.35	1.40 (4.63)
**Southeast**	5.77	2.75	0.01	17.89	88.33	0.44 (1.38)
**South**	6.22	3.17	0.02	16.33	87.37	0.02 (3.07)
**Brazil**	11.11	5.26	0.04	31.36	89.58	1.05 (2.82)

SBE, moderate, severe events, and mortality were calculated by 100,000 inhabitants.

Availability of hospitals with ICU and antivenom is an index which represents the amount of ICU beds per 100,000 inhabitants.

**Fig 2:**
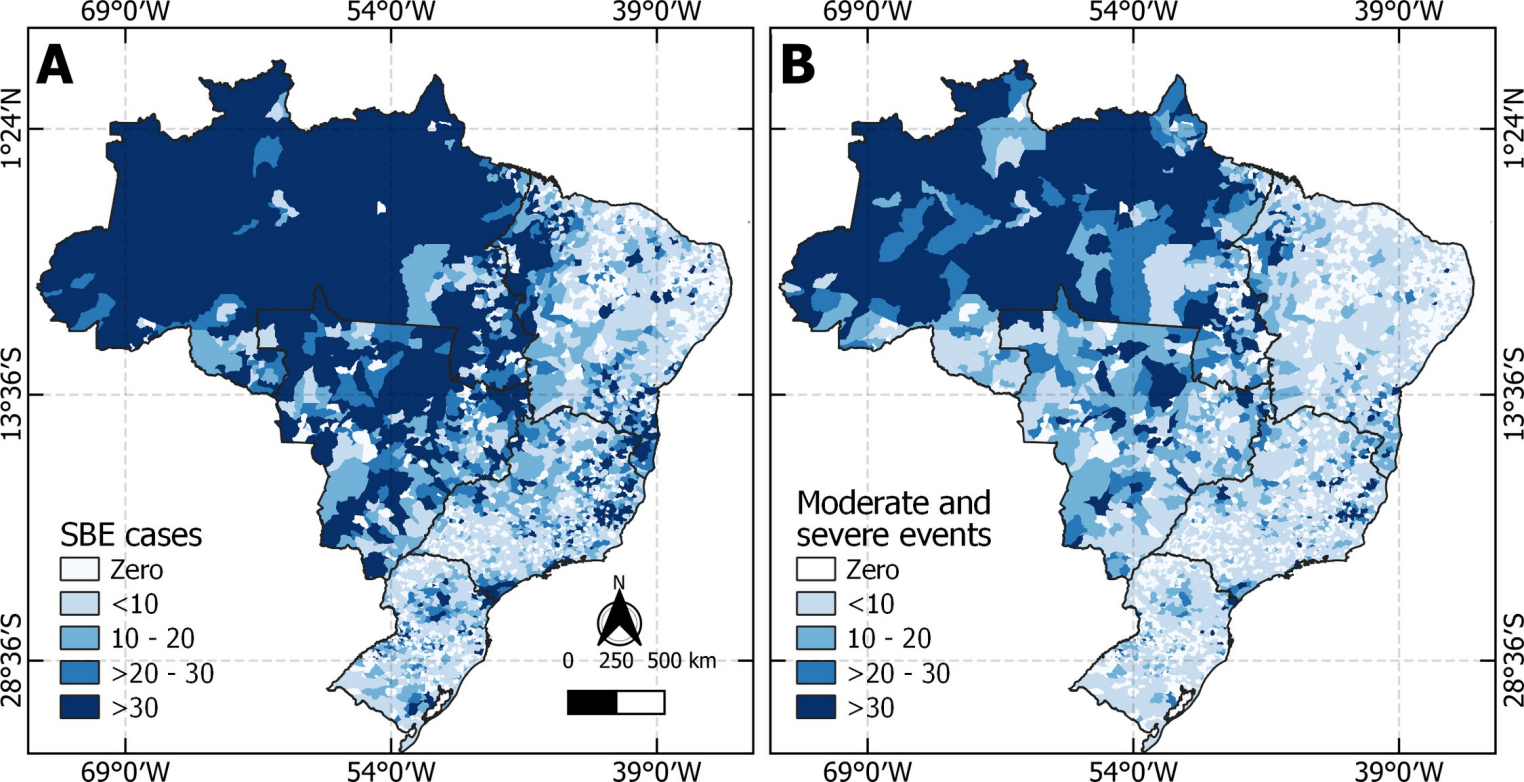
SBE (A) and moderate and severe events (B) by 100,000 inhabitants. Source for base layer: Instituto Brasileiro de Geografia e Estatística [17].

**Fig 3:**
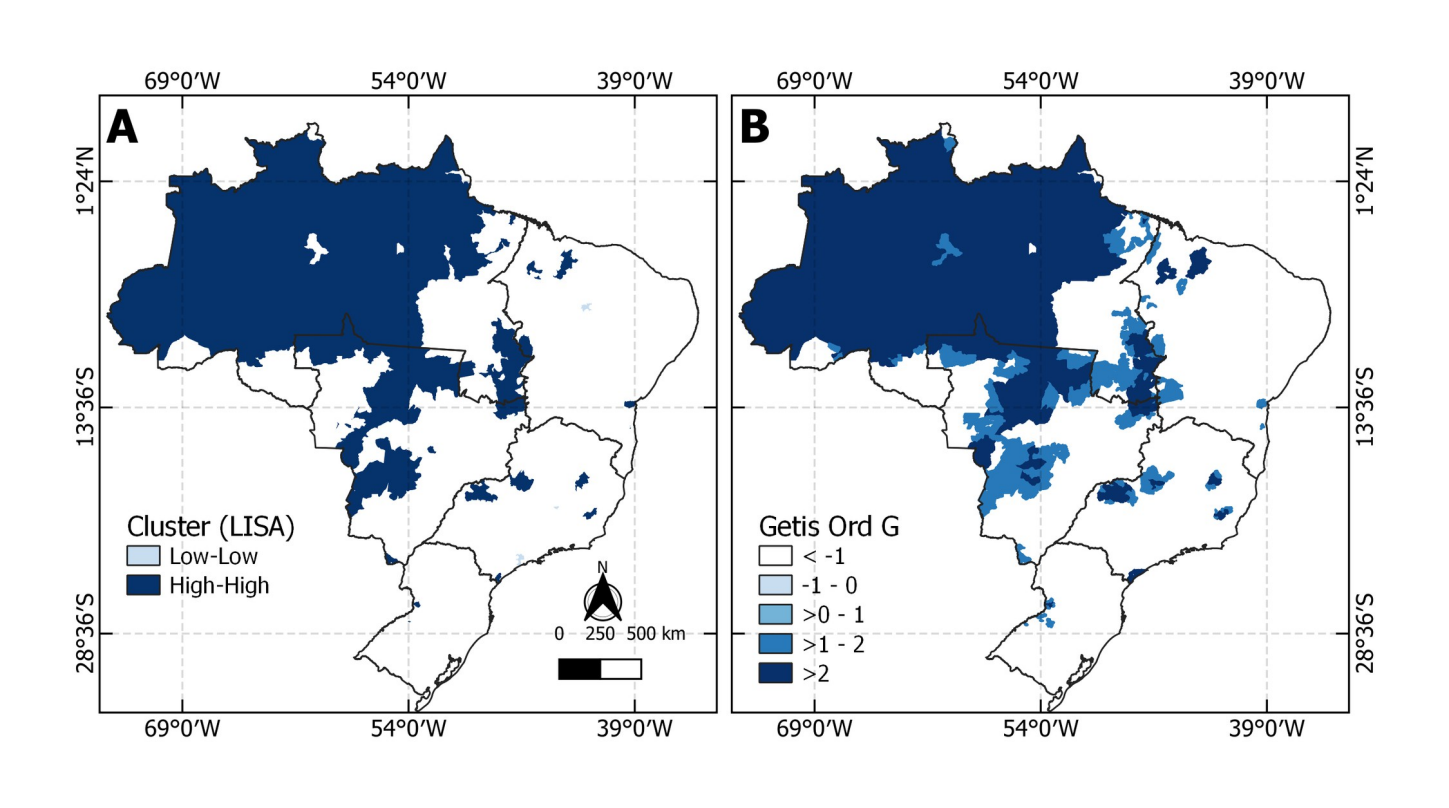
A) Local Indicator of Spatial Autocorrelation (LISA) showing clusters regarding moderate and severe rates of snakebite envenoming. B) Getis Ord G scores presenting the location of hotspots based on rates of moderate and severe cases. Source for base layer: Instituto Brasileiro de Geografia e Estatística [17].

Fig 2 presents the incidence of SBE, and moderate and severe events in each municipality per 100 thousand population. Notably, the North region, where most of the Amazon Forest is located (Fig 3A), is characterized by both the highest rates of SBE and the highest rates of moderate and severe events. The northeast part of the Midwest region presented the same behavior. In the Southeast region, the Northeast portion of Minas Gerais demonstrated a high incidence of SBE per population. In Fig B, it is possible to observe that cases classified as moderate or severe are concentrated in the North region.

Panel A in Fig 3 shows the clusters high-high where regions with high incidence of moderate and severe cases are surrounded by neighbors with high incidence of moderate and severe cases. Low-low clusters are the opposite, that is, municipalities with low incidence of moderate and severe cases are surrounded by municipalities with low rates.

**Fig 4:**
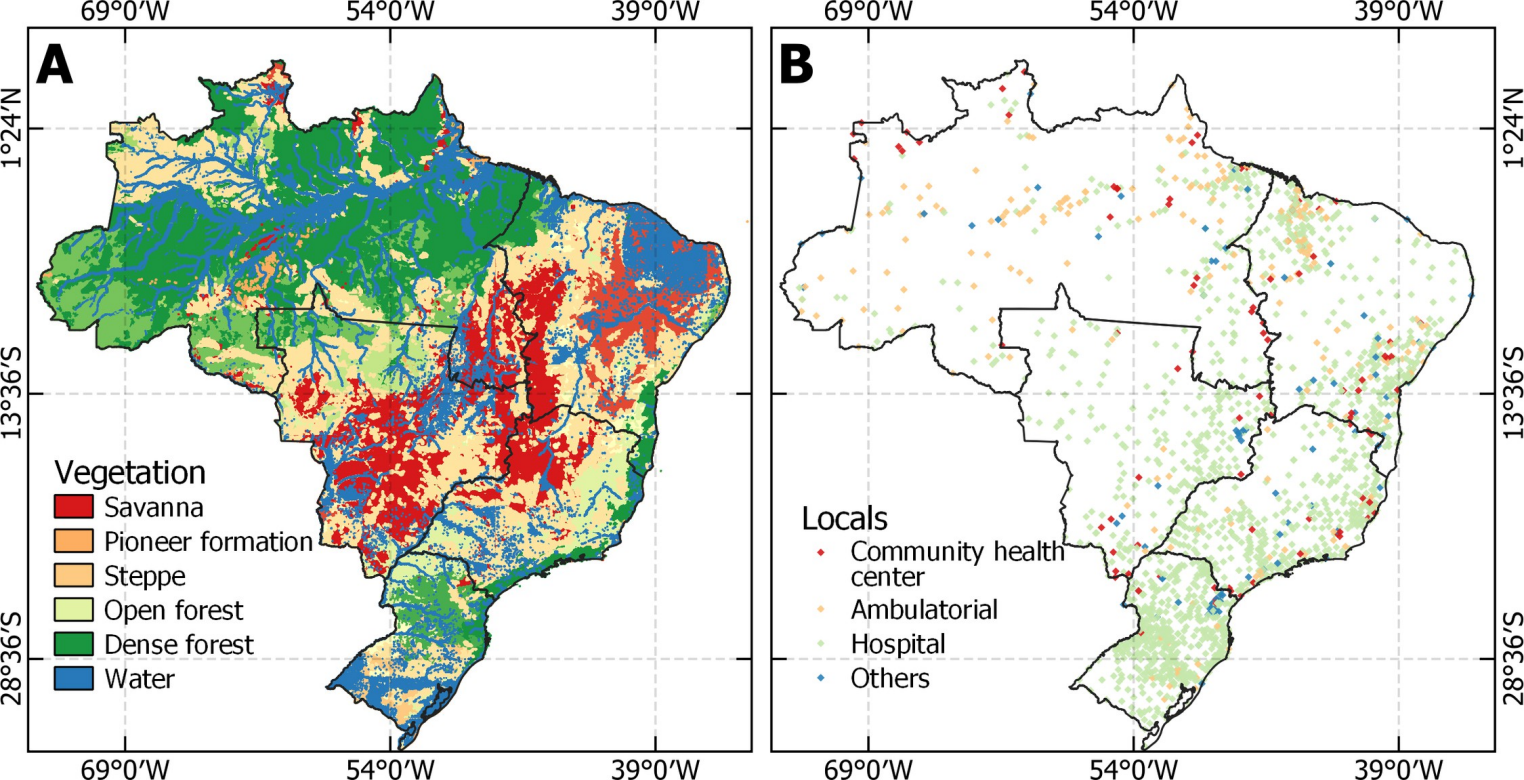
Location and distribution of antivenom health facilities. Source for base layer: Instituto Brasileiro de Geografia e Estatística [17].

**Fig 5:**
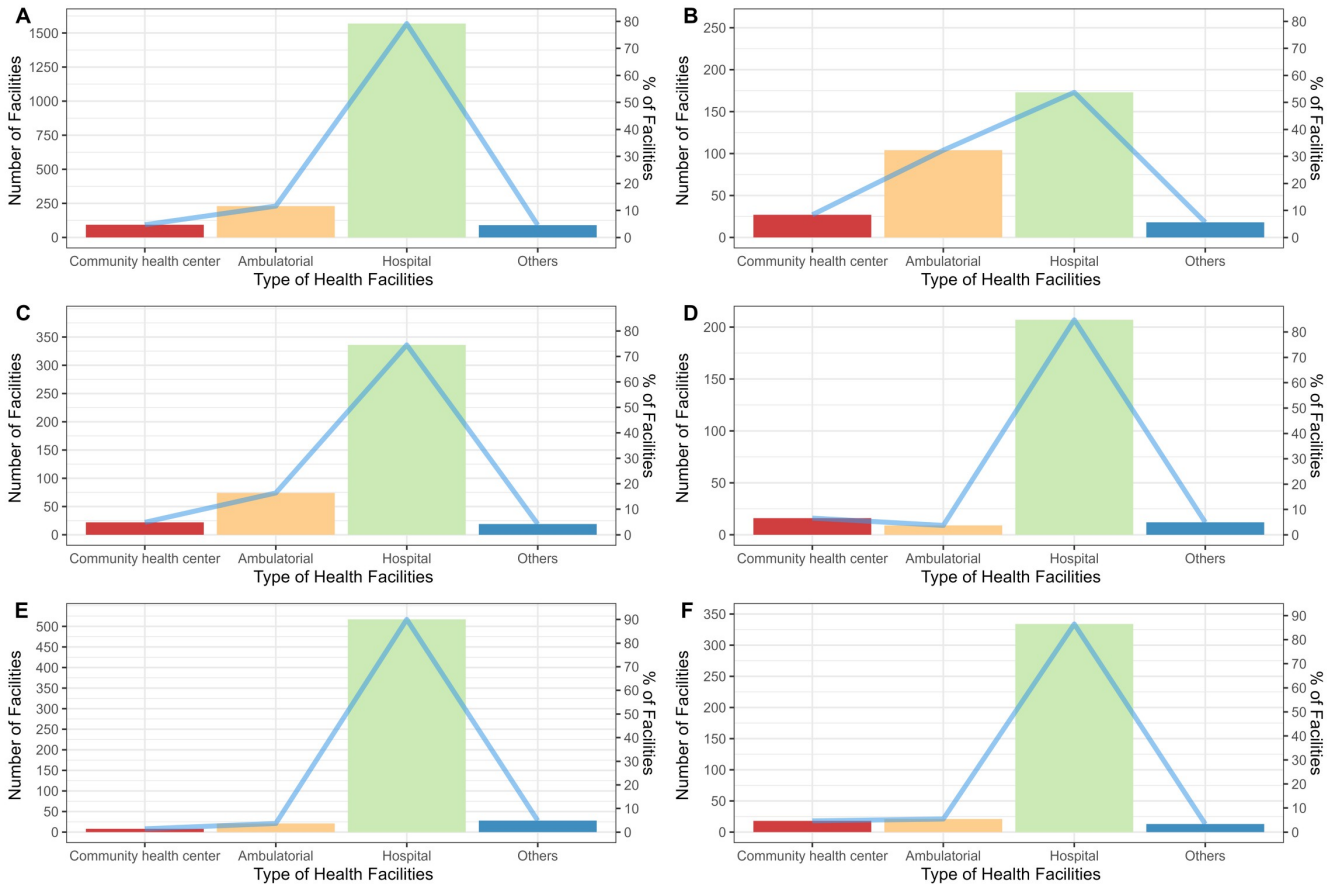
Panels A represents the number and percentage of health facilities with antivenom in Brazil, and panels B, C, D, E, and F represent the regions North, Northeast, Midwest, Southeast, and South respectively.

Fig 4 presents the location of facilities with antivenom in Brazil, and Fig 5 shows that antivenom is mostly available in hospitals in Brazil, followed by ambulatorial and community health centers. It is notable to mention that the ambulatorial and CHC decentralize antivenom services in the North region, while the South and Southeast regions are more focused on hospitals.

**Fig 6:**
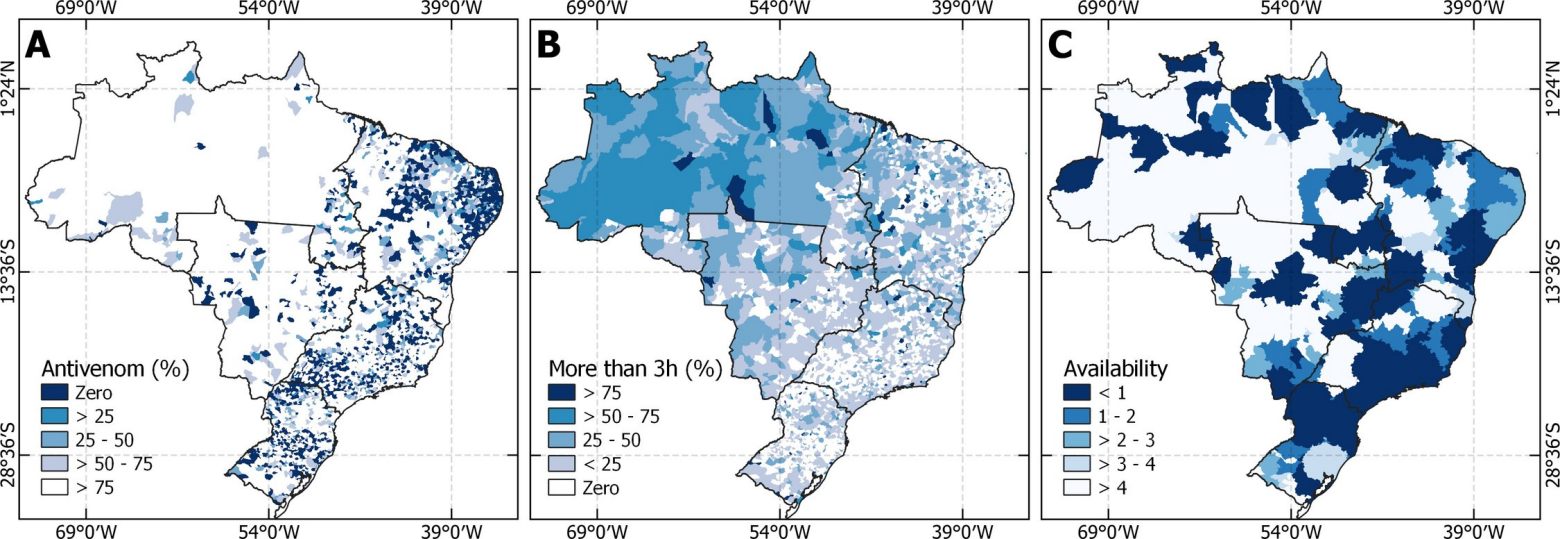
A) Percentage of cases that antivenom was used. B) Percentage of cases that took more than 3 hours to reach a health facility to care. C) Availability of ICU beds in health facilities supplied with antivenom per 100,000 population. Source for base layer: Instituto Brasileiro de Geografia e Estatística [17].

Panel A in Fig 6 shows the percentage of SBE cases where antivenom therapy was used. In the North region, where most SBE cases occurred, more than 75% of cases received antivenom. Other areas of the country used antivenom in a lower proportion of cases. Panel B demonstrates that at least 25% of SBE victims in the North region took more than three hours to seek care. Finally, Panel C shows the results of the 2SFCA weighting of the existing ICU beds in facilities with antivenom, by the population close to the facility. The value plotted in the map is accessibility index, highlighting the number of ICU beds available per 100 thousand population. Despite the large number of health facilities in the South, Southeast and Northeast regions, the large population contributes to a low accessibility index. In the North and Midwest, the low population density results in the opposite pattern.

**Fig 7:**
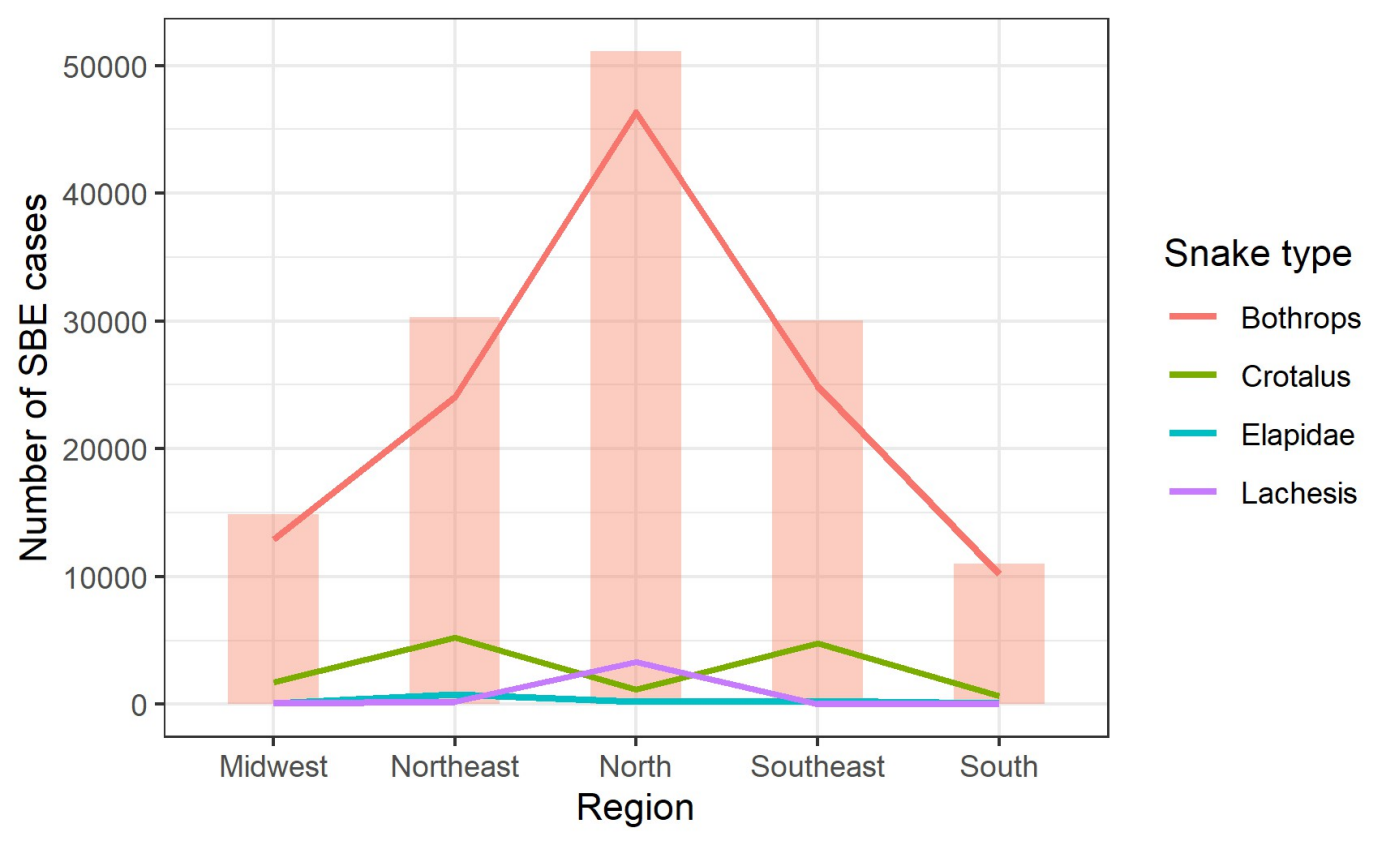
Distribution of snakebite accidents in each region and by genre/family.

Fig 7 shows the distribution of SBE accidents across the country. It is notable that the genre *Bothrops* is responsible for the majority cases. In addition, *Bothrops* is the most frequent in the North region. Elapidae is the least frequent family of snakes causing accidents in Brazil.

Table 4 shows the results of the two regression analyses used the evaluate the association of ou socio-economic predictos and the number of moderate and severe events caused by SBE. As GWPR presented the highest pseudo R^2^ (0.634), and lowest AIC (85,034.51) and Moran’s I residuals (0.003), this model had the best performance. In the model, life expectancy, young population structure, income, inequality, schooling, school attendance, sanitation, electricity, occupation, formal work, more than three hours to seek care, and availability of ICU beds in facilities with antivenom explained 63.4% of variance of moderate and severe events. The GLM was not selected because it generated evidence of misspecification attributed to high values in the residuals.

**Table 4:** Performance comparison between Generalized Linear Models using Poisson regression and Geographically Weighted Poisson Regression.

Variables	GLM			GWPR		
	Estimate	Standard error	P-value	1^st^ Quantile	Median	3^rd^ Quantile
Constant	1.818	0.006	<0.01	1.254	1.869	2.285
Life expectancy	0.243	0.006	<0.01	0.079	0.148	0.253
Young population	0.360	0.006	<0.01	-0.269	0.050	0.269
Income	-0.690	0.010	<0.01	-0.754	-0.401	-0.151
Inequality	0.208	0.004	<0.01	0.320	0.402	0.480
Schooling	-0.092	0.010	<0.01	-0.800	-0.462	-0.190
School attendance	0.004	0.005	0.508	-0.402	-0.261	-0.138
Sanitation	-0.600	0.014	<0.01	-0.472	0.227	1.092
Electricity	0.215	0.011	<0.01	-1.386	-0.420	0.143
Occupation	0.092	0.003	<0.01	0.075	0.236	0.456
Formal work	0.206	0.005	<0.01	-0.097	0.074	0.341
More than 3h	0.425	0.003	<0.01	0.276	0.365	0.452
Availability	-0.029	0.002	<0.01	-0.593	-0.165	0.015
Moran’s I		0.213		0.028		
Moran’s Residuals		0.288		0.003		
AIC		125,016.10		85,034.51		
Pseudo R^2^		0.461		0.634		

**Fig 8:**
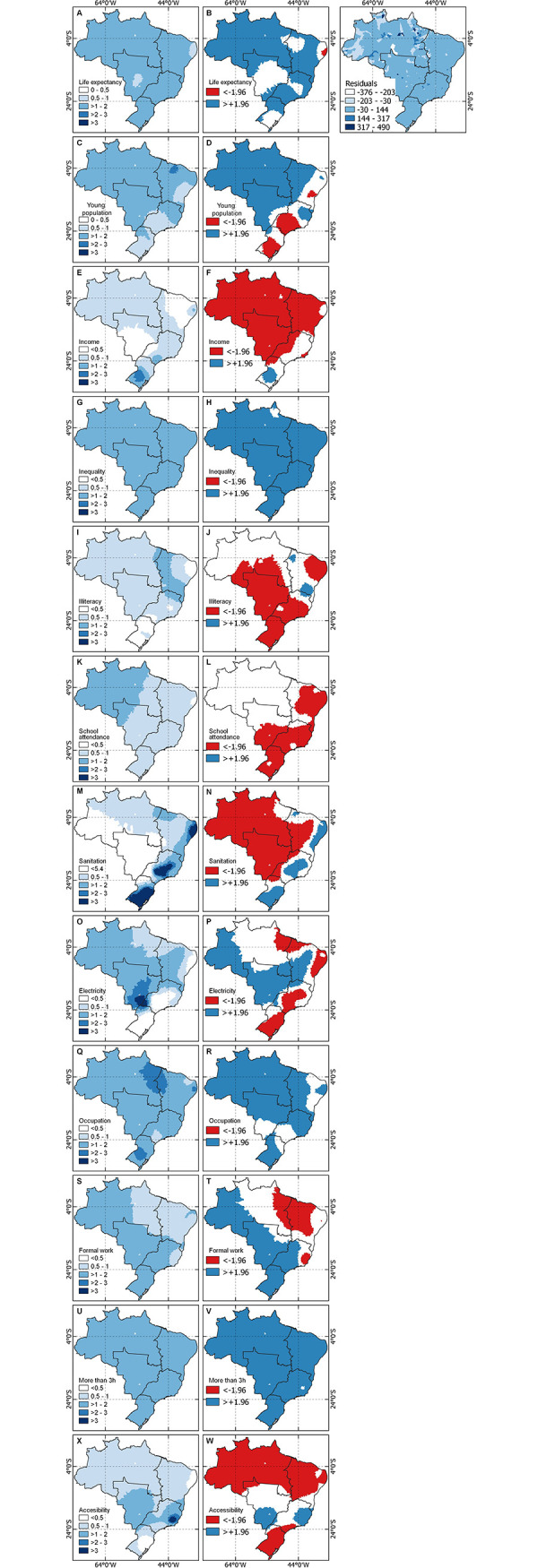
Association of various sociodemographic and access to care indicators with moderate or severe outcomes after SBE. Left) Magnitude of association in exponential values. Right) Statistical significance of association according to T-values. Source for base layer: Instituto Brasileiro de Geografia e Estatística [17].

Fig 8 represents the results of the GWPR, with the coefficients and significance (T-value) of each predictor. The diverging palette in the first column highlights the magnitude of the association between the regression coefficient and the moderate and severe event outcome. The second column of the Fig presents the regions in which the values obtained by a coefficient are statistically significant and thus contributing to the variance in SBE outcomes. The blue regions in the second column are positively associated with the outcome measure and the red regions are negatively associated.

Positive indicators were life expectancy, inequality, occupation, and more than three hours to reach care. On the other hand, negative indicators were income and illiteracy. The rest of the indicators like young population structure, electricity, formal work, sanitation, and accessibility demonstrated a mixed pattern, suggesting that SBE is a complex phenomenon with multiple variables underlying rates of these events that vary across the country.

## Discussion

The results of this study demonstrate regional disparities in SBE metrics and outcomes in Brazil with the Amazon Region disproportionately affected. This is the first study in Brazil analyzing the geospatial distribution of SBE moderate and severe events and the association with sociodemographic variables and access to care. Previous studies have evaluated the geographical distribution of SBE in Brazil and globally, but this is the first analysis of this sort trying to identify the healthcare gaps and how these are related to sociodemographic indicators [11,32].

Our results also demonstrate that inadequate availability is part of the problem causing poor outcomes post SBE. Despite the North having an apparent great availability of ICU beds at facilities that administer antivenom, the region still experiences high rates of moderate to severe SBE events. This can be attributed to the fact that our analysis only accounted for resources specific to snakebite envenoming, rather than considering overall health resources in the region, which still ranks lowest in terms of overall health assistance [26]. Additionally, as the North region presents a low population density (4.12 inhabitants/km^2^), this can also contribute to a higher ratio of availability compared to regions with higher density, such as the Southeast with over 87 inhabitants/km^2^ [15]. Therefore, as the time-dependency of antivenom administration is a core tenant of SBE treatment, the development of new strategies to improve the timeliness of access to SBE care in Brazil, especially in remote areas, is essential.

Our results revealed that multiple indicators relating to demographics, economics, occupation, education, and sanitation/electricity were statistically associated with moderate and severe outcomes after SBE. In the North region, a young population structure was positively related to frequency of SBE and rate of severe outcomes. Young populations, who are more commonly social and involved in outdoor activities, may be more likely to get exposed to SBE [33]. This association likely holds especially true in the North as SBE in the Amazon are predominantly associated with work in the field, such as agriculture [34,35].

Additionally, in much of the country, low income was associated with increased frequency SBE. In Latin America, both indigenous groups and migrant populations, who are more likely to live in poverty, account for a disproportionate number of SBEs with a prevalence at least 7.5 times higher [36]. Further, members of many indigenous communities may choose to first seek care from traditional healers after SBE, leading to delays in the definitive care with antivenom and increased rates of severe outcomes [37].

Speaking of SBE in Latin America, a study conducted between 2010 to 2016 revealed the overall incidence of snakebites is 6.34 per 100,000 population, while the mortality is 0.03 per 100,000 population [38], contrasting the incidence of our study in which presented a higher number with 11.11 per 100,000, and a mortality of 0.04 per 100,000. Colombia, a country neighboring Brazil, presented mortality ranging from 0.05 to 0.09 per 100,000, with annual incidences between 7.0–9.7 cases per 100,000 inhabitants according to a study from 2008 to 2016 [39], where the majority of the cases happened in regions of border with Brazil probably because of the Amazon Region.

Another variable inversely associated with SBE events and outcomes was education. Proper education is fundamental to obtaining knowledge regarding SBE prevention [40,41]. Further, patients of lower educational status may be late to recognize serious clinical symptoms and resistant to seeking medical assistance, instead engaging in ineffective and harmful self-care practices [9].

In addition, sanitation was associated with SBE events. According to the World Health Organization’s Executive Summary on SBE, inadequate sanitation and poorly constructed housing increase the risk of SBE [42]. For example, communal latrines may attract rodents, drawing venomous snakes near the population [42].

The ability of these indicators to predict adverse SBE outcomes was generally only statistically significant in the Northern part of the county. Fewer predictors were relevant in explaining the SBE negative outcome rates in the South, Southeast, and Northeast regions. In these regions, only a few municipalities were identified as presenting with statistically significant variables and the regions in which they did so were often small. In the South, outcomes in areas with poor indicators were not significantly affected. Our results suggest that differences in access to care help explain why such variables only affect outcomes in some portions of the country.

We found that, in the North region and bordering areas of the Midwest and Northeast, more than three hours to reach care was significantly associated with increased rates of severe SBE outcomes. People in North of Brazil, generally with poor indicators, also face long travel times, delays in care, and smaller hospitals with antivenom availability. In these areas, which overlap with the expanse of the Amazon Rainforest, considerable difficulties with travel exist and many people rely on river transportation to reach city hospitals [3]. Our results demonstrate that inadequate access to care is the major problem driving poor outcomes post-SBE.

Since the World Health Organization declared a novel strategy that aims to reduce 50% snakebite deaths by 2030 via detecting the variables that affect severe cases [41], here we presented that sociodemographic indicators and adequate time for access to care must be prioritized in order to improve SBE outcomes. Previous work repeatedly demonstrated that delays in care greater than six hours after SBE are associated with increased likelihood of severe systemic envenomation [6]. The results of our work, which demonstrate that requiring more than three hours to reach to care is associated with an increase in the number of moderate and severe events, suggests that the treatment of SBE may even be more time sensitive than previously reported.

Ultimately, it is important to note that different species of snake bites may lead to different outcomes due to their particularities. In Brazil, snakes of public health interest belong to the Viperidae and Elapidae families, and antivenom is necessary for bites from the genera *Bothrops*, *Crotalus*, *Lachesis*, and *Micrurus*. Notably, 72–83.8% of snakebite incidents requiring antivenom are caused by the *Bothrops* genus and about 8% by *Crotalus* [11,43]. For example, *Bothrops jararaca*, endemic in the southeast region of Brazil, has venom components belonging to metalloproteinases, serine proteinases, phospholipase A2, and biologically active peptides that cause local and systemic effects, edema, inflammation, myonecrosis, hemorrhage, hemostasis, renal failure, and shock [43]. On the other hand, *Crotalus durissus* (South American rattlesnakes) reported in the Northeast, Midwest and South of the Amazonian Forest in Brazil [44], have four toxins in their venom—crotoxin, crotamine, gyroxin, and convulxin—which may cause severe neurotoxic symptoms and acute nephrotoxicity, hemotoxicity, cardiovascular and respiratory disturbances [45].

This work demonstrates that to reduce the number of moderate and severe outcomes associated with SBE, we must improve the accessibility of care and, importantly, antivenom. Additionally, it is also important to evaluate the socioeconomic variables because they may play an important role regarding the accessibility to care. One potential solution to optimize SBE care delivery, specifically antivenom distribution, to Brazil’s Amazon is to leverage the existing community healthcare centers (CHCs) network [35]. Currently, antivenom is produced in Brazil by national government agencies, but widespread distribution, especially to remote regions, is restricted. To reduce SBE-related morbidity and mortality, it is crucial that health facilities are reliably supplied with antivenom and are capable of its safe administration [46]. Brazil’s healthcare system, Sistema Único de Saúde (SUS), one of the largest public healthcare systems in the world, emphasizes community empowerment in providing comprehensive primary care [47]. The SUS Family Health Strategy, the system’s primary care model, covers about 90% of Brazil’s municipalities, 70% of the population, and reaches most of the country’s remote regions including the Amazon. Using CHCs to address antivenom delivery challenges in low resource settings has been proposed; however, to date, there have been no studies evaluating the feasibility of the proposal or attempts at initiating change [35,48,49]. We hope that the results of our work, which highlight the devastating impact of inadequate access to care on SBE outcomes, inform public health discussions surrounding improving the response to this neglected tropical disease.

### Limitations

Although the SINAN database is intended to be comprehensive and accurate, it is susceptible to issues regarding reliability and consistency of data. Additionally, provider-level reporting of SBE relies on accurate recognition and diagnosis. Thus, the resultant underreporting may not capture the scope of this important disease. Second, using a 120km radius to define the catchment area of health facilities may not be uniform considering rainforest, rural areas, and the quality of the roads across the country is not equivalent, meaning that patients may face different difficulties to access a health facility with antivenom. In addition, patients in the Amazon Region frequently rely on boats to access a health facility, and the speed of a boat is different from a car. And finally, we conducted a Poisson regression to verify the predictors related to moderate and severe events when taking the spatiality into the model, in which the model could be affected by zero-inflated issues from municipalities with no existing or reported cases.

### Conclusion

In Brazil, the number of moderate and severe events caused by SBE vary across the country and are correlated with sociodemographic indicators such as health, education, economics, occupation, and sanitation, as well as access to care. Innovative approaches to increasing access to antivenom such as leveraging existing community healthcare centers, especially in the Northern portion of the country, may help reduce unfavorable SBE outcomes.
